# Nonmotor Symptoms Affect Sleep Quality in Early-Stage Parkinson's Disease Patients With or Without Cognitive Dysfunction

**DOI:** 10.3389/fneur.2020.00292

**Published:** 2020-04-21

**Authors:** Jun Zhu, Min Zhong, Jun Yan, Xu Jiang, Zhuang Wu, Yang Pan, Bo Shen, Lili Zhang, Jingde Dong, Li Zhang

**Affiliations:** ^1^Nanjing Brain Hospital Affiliated to Nanjing Medical University, Nanjing, China; ^2^Nanjing Medical University, Nanjing, China

**Keywords:** Parkinson's disease, nonmotor symptoms, sleep quality, polysomnography, early stage

## Abstract

**Purpose:** Parkinson's disease (PD) patients frequently present with sleep disorders. This study was designed to assess the impact of nonmotor symptoms (NMSs) on sleep quality in early-stage PD patients with and without cognitive dysfunction.

**Materials and Methods:** A sample of 389 early-stage PD patients (modified Hoehn and Yahr score ≤ 2.5, duration ≤ 5 years) was recruited for the present study. The Non-Motor Symptoms Questionnaire (NMS-Quest) was used to screen for global NMSs. Depressive symptoms were assessed using the Hamilton Rating Scale for Depression (HAMD). PD motor symptoms were measured with the Unified PD Rating Scale (UPDRS), part III. The Montreal Cognitive Assessment (MoCA) was used to evaluate global cognitive status, and the PD Sleep Scale (PDSS) was used to quantify sleep quality. Polysomnography (PSG) was used for objective assessment of sleep.

**Results:** In our sample, approximately one-quarter of the PD patients suffered from sleep disturbances (23.7%). Our results also confirmed the high prevalence of cognitive dysfunction in patients with PD (39.8%). In patients with cognitive dysfunction, higher percentage of sleep disorders (34.8 vs. 16.2%, *P* < 0.01) was observed. They also with lower PDSS score, sleep efficiency (SE) and longer sleep lantency (SL) and wake after sleep onset (WASO) (All *P* < 0.05). In total, the patients who suffered from NMSs, such as depressive symptoms, anxiety symptoms, urinary tract symptoms and hallucinations/delusions, had poorer sleep quality. Better cognition may predict better sleep quality. In patients with cognitive dysfunction, the NMS-Hallucinations/delusions score was the most important risk factor for sleep disorders. In patients without cognitive dysfunction, NMSs such as anxiety and cognition and medication were related to sleep disorder.

**Conclusions:** NMSs in early-stage PD are highly associated with and are determinants of subjective sleep quality. Future studies should focus on elucidating the pathophysiology of these symptoms.

## Introduction

Parkinson's disease (PD) is the second most common neurodegenerative disease in the Chinese population aged over 65 years (1). PD is mainly defined by motor symptoms such as tremor, rigidity, bradykinesia, and postural instability. Damage to the dopaminergic system plays an important role in the motor symptoms of PD patients. However, the pathological course of PD has been found to involve the serotoninergic, noradrenergic, and cholinergic systems in addition to the dopaminergic system (2). Dysfunction in these nondopaminergic systems may play a crucial role in the development of nonmotor symptoms (NMSs), including neuropsychiatric manifestations, autonomic and sensory dysfunctions, and sleep disorders (3). NMSs of PD can precede the emergence of typical motor symptoms and the diagnosis of PD by several years.

Sleep disturbance affects 60–98% of patients with PD (4). As one of the most common nonmotor features of PD, sleep disturbance exerts a detrimental effect on health-related quality of life (5). In PD, sleep problems include not only difficulties with falling asleep and staying asleep but also difficulties with sleep fragmentation, nocturnal akinesia, restless legs syndrome (RLS), rapid eye movement sleep behavior disorder (RBD), obstructive sleep apnea syndromes, nocturia and excessive daytime sleepiness (EDS) (4). The pathogenic mechanisms underlying sleep disorders in PD are not completely understood. First, these disorders may be related to the underlying neuropathology of and neurochemical changes in PD. Abnormalities in dopamine and/or other neurotransmitter pathways, such as norepinephrine, serotonin, dopamine and GABA (6), may be responsible for sleep disorders in PD patients. Second, patients with PD have nighttime motor symptoms, including rigidity, tremor, and poor bed mobility. In addition, PD patients are susceptible to age-related problems such as nocturia, circadian changes and joint pain.

Cognitive impairment is common in PD, and several studies of patients with incident PD affirm that cognitive dysfunction is not solely a complication of advanced disease. More subtle cognitive dysfunction appears in the early stages and affects mainly the attention, executive function, verbal fluency, and visuospatial domains (7).

The etiology of nocturnal sleep disturbances in PD is likely multifactorial. The various degrees of disability, motor symptoms and NMSs and complications of therapy may have differential impacts on sleep quality (8, 9). Well-known NMSs that can affect sleep quality may include neuropsychiatric symptoms; depression; anxiety; autonomic nervous system dysfunction, such as nocturia; cardiac sympathetic denervation; and excessive sweating. However, there is a lack of reports on the association between NMSs and sleep quality in patients with early-stage PD with or without cognitive impairment.

In the current observational study, we sought to determine the prevalence of sleep disorders and cognitive impairment and their associated factors in a group of patients with early-stage PD. The relationships between sleep quality and other NMSs were also investigated.

## Materials and Methods

### Population

A consecutive series of 389 patients were recruited from neurological clinics in the Department of Neurology, Nanjing Brain Hospital, Nanjing Medical University between January 2014 and October 2019. The diagnosis of PD was based on the United Kingdom Parkinson Disease Society Brain Bank Clinical Diagnostic Criteria (10). Patients with diagnoses of progressive supranuclear palsy, multiple system atrophy, corticobasal degeneration, and other forms of atypical parkinsonism were excluded. Patients with severe illness, acute systemic disorder or injury, acute cerebral ischemia, encephalitis or psychosis were also excluded. Individuals were excluded if they did not have sufficient comprehension to complete the study questionnaire. None of the enrolled patients took related sleep medications, such as benzodiazepines, phenolbarbital, melatonin, tricyclic antidepressants (TCAs) and selective serotonin reuptake inhibitors (SSRIS).

### Procedures

A structured questionnaire was utilized to obtain the demographic information and clinical characteristics of each patient, including age, sex, disease duration, education level, and use of antiparkinsonian drugs. Levodopa equivalent doses (LEDs) were calculated according to previously published recommendations. The questionnaires were administered by a trained specialist during face-to-face interviews. This study was approved by the Ethics Committee of the Brain Hospital Affiliated with Nanjing Medical University (Ethics approval number: 2015-KY-041), and the study was performed in accordance with the principles of the Declaration of Helsinki. After receiving a complete explanation of the study, all participants signed a written informed consent prior to the initiation of the study.

The Unified PD Rating Scale (UPDRS), part III, was used to assess motor disability (11). For the analysis, the patients were designated as “early-stage” on the basis of the modified Hoehn and Yahr score (mH&Y) (12) and disease duration: (1) mH&Y stage ≤ 2.5; (2) disease duration <5 years (calculated as the period, in years, between the first motor symptom experienced by the patient and the date of assessment) (13). The Non-Motor Symptoms Questionnaire (NMS-Quest) was used to screen for global NMSs and covers nine dimensions: cardiovascular, sleep, depression/anxiety/anhedonia, apathy/attention/memory, gastrointestinal symptoms, urinary symptoms, sexual function, and miscellaneous (14). Cognitive function was evaluated using the Montreal Cognitive Assessment (MoCA) (15). The MoCA is a suitably accurate, brief test for screening all levels of cognition; the optimal cutoff score is 26 for PD with cognitive dysfunction (16). The PD Sleep Scale (PDSS) consists of 15 items examining sleep disturbances and nocturnal problems associated with PD (17) and was used to assess subjective sleep quality. Scores of 105 or more suggest normal sleep (18). We used the 24-item Hamilton Depression Scale (HAMD) (19), a validated tool recommended by the American Academy of Neurology, to screen for depression in PD (20). A cutoff score of 20 or higher indicates the presence of depression (21). The 24 items are grouped into 7 domains: (1) anxiety/somatization (6 items: psychic anxiety, somatic anxiety, gastrointestinal symptoms, hypochondriasis, insight, general somatic symptoms), (2) weight loss, (3) mental disorder (6 items: feelings of guilt, suicide, agitation, depersonalization and derealization, paranoid symptoms, obsessional symptoms), (4) diurnal variation, (5) retardation symptoms (4 items: depressed mood, work and interests, retardation, genital symptoms), (6) sleep disturbance (3 items: early, middle, and late insomnia), and (7) hopelessness (3 items: helplessness, hopelessness, worthlessness). The 14-item Hamilton Anxiety Rating Scale (HAMA) was administered to examine anxiety (22).

### Polysomnography (PSG)

The full-night, attended, laboratory-based video-PSG was recorded on a Compumedics Grael-HD 64 (Compumedics Grael-HD, Australia) polygraph. The time of the PSG recording was fixed (between 23:00 and 07:00). Sleep parameters such as sleep latency (SL), wake after sleep onset (WASO) and sleep efficiency (SE) were used as objective indicators for evaluating sleep. SL ≥30 min, WASO ≥60 min and sleep efficiency <85% on PSG as well as PDSS <105 were defined as sleep disorders. NREM sleep stage 1 (N1) time (min), NREM sleep stage 3 (N3) time (min), total sleep time (min), the apnea-hypopnea index (AHI) and periodic limb movement index (PLMI) were also used to compare the sleep quality of patients with/without cognitive impairment.

### Statistical Analysis

All continuous data are presented as the mean ± standard deviation (SD), and categorical variables are shown as percentages. Student's *t*-test was applied for comparisons of continuous data, and chi-square tests were performed to examine differences in categorical variables between patients with and without sleep disorders. Continuous variables, including the total scores for the NMS-Quest, the HAMD and the PDSS, as well as the scores for each domain of the NMS-Quest and the HAMD, were compared using one-way analyses of covariance (ANCOVAs) after adjustments for confounding factors, including sex, age, PD duration, UPDRS-III score, mH&Y stage, and LED. A multivariate analysis using a forward binary logistic regression model with sleep disorders as the dependent variable and the above significant disease characteristics as independent covariables was used to explore the potential clinical factors that may be related to sleep disorders. Other factors, such as age, which is clinically considered independent variables that is closely related to sleep disorders, was also included. All analyses were performed with SPSS 19.0 (IBM Corp., Armonk, NY). *P* < 0.05 was considered statistically significant.

## Results

### Sociodemographic and Clinical Variables of Patients

In total, 389 subjects with PD, including 247 men (63.5%) and 142 women (36.5%), were enrolled in this cross-sectional study. The patients' demographic characteristics are presented in Table 1. The mean age was 63.88 ± 9.06 years, and the mean age at disease onset was 58.57 ± 9.19 years. The mean duration of the disease was 3.32 ± 2.12 years. The mean mH&Y score was 1.46 ± 0.55, and the mean UPDRS part III motor score was 18.16 ± 9.15. The patient cohort had a mean total PDSS score of 121.32 ± 19.20. In total, 92 of the 389 (23.7%) patients reported significant sleep disturbances. The mean MoCA score was 25.41 ± 2.70. Using a cutoff of 26 points on the MoCA, 155 (39.8%) patients were found to have cognitive dysfunction. The average HAMD score was 10.82 ± 8.25, the average PD-NMS score was 9.37 ± 4.83, and the scores for each domain are also presented.

**Table 1:** Sociodemographic and clinical variables of patients.

**Patients**	***N***	**Range**
Sex		
Male	247	63.5%
Female	142	36.5%
Age, y, mean ± SD	63.88 ± 9.06	41–73
Duration, y, mean ± SD	3.32 ± 2.12	0.5–5
UPDRS-III, mean ± SD	18.16 ± 9.15	3–38
mH&Y stage, mean ± SD	1.46 ± 0.55	1–2.5
1	124	31.9%
1.5	118	30.3%
2	90	23.1%
2.5	57	14.7%
NMS-Quest, mean ± SD	9.37 ± 4.83	0–14
Gastrointestinal tract symptoms, mean ± SD	1.82 ± 1.33	0–5
Urinary tract symptoms, mean ± SD	0.83 ± 0.85	0–2
Apathy/attention/memory, mean ± SD	1.57 ± 1.06	0–3
Depression/anxiety/anhedonia, mean ± SD	0.81 ± 0.85	0–2
Sexual function, mean ± SD	0.51 ± 0.84	0–2
Cardiovascular symptoms, mean ± SD	0.44 ± 0.62	0–2
Sleep, mean ± SD	1.88 ± 1.34	0–5
Miscellaneous, mean ± SD	1.00 ± 0.92	0–3
PDSS, mean ± SD	121.32 ±19.20	51–150
Sleep quality, mean ± SD	24.38 ± 4.67	14–28
Nocturnal restlessness, mean ± SD	14.47 ± 4.39	7–20
Nocturnal psychosis, mean ± SD	15.65 ± 4.10	8–20
Nocturia, mean ± SD	7.24 ± 2.38	3–10
Nocturnal motor symptoms, mean ± SD	34.79 ± 7.09	19–37
Daytime somnolence, mean ± SD	15.03 ± 3.45	9–20
Without Sleep disorders	297	76.3%
With Sleep disorders	92	23.7%
Sleep lantency, min, mean ± SD	26.79 ± 22.17	1–138
Wake after sleep onset, min, mean ± SD	48.34 ± 38.49	0–247
Sleep efficiency, %	89.23 ± 56.87	52–98
HAMA, mean ± SD	9.17 ± 6.94	0–36
HAMD, mean ± SD	10.82 ± 8.25	0–49
Anxiety/somatization, mean ± SD	2.63 ± 2.43	0–13
Loss of weight, mean ± SD	0.39 ± 0.66	0–3
Mental disorder, mean ± SD	1.28 ± 1.94	0–16
Diurnal variation, mean ± SD	0.21 ± 0.43	0–3
Retardation, mean ± SD	2.43 ± 2.06	0–11
Sleep disturbance, mean ± SD	1.68 ± 1.93	0–11
Hopelessness, mean ± SD	2.19 ± 1.86	0–10
MoCA, mean ± SD	25.41 ± 2.70	20–30
Visuospatial skills/executive function, mean ± SD	3.71 ± 1.21	0–5
Naming, mean ± SD	2.75 ± 0.55	0–3
Attention, mean ± SD	5.49 ± 0.76	3–7
Language, mean ± SD	2.62 ± 0.63	0–4
Abstraction, mean ± SD	1.67 ± 0.57	0–2
Delayed memory, mean ± SD	3.31 ± 1.40	0–5
Orientation, mean ± SD	5.86 ± 0.42	4–6
Without cognitive dysfunction	234	60.2%
With cognitive dysfunction	155	39.8%
LED, mg, mean ± SD	357.38 ± 327.60	0–779

*SD, standard deviation; PD, Parkinson's disease; UPDRS-III, Unified Parkinson's Disease Rating Scale part III; mH&Y stage, modified Hoehn and Yahr stage NMS-Quest, Non-Motor Symptoms Questionnaire; PDSS, Parkinson's Disease Sleep Scale; HAMA, Hamilton Anxiety Rating Scale; HAMD, Hamilton Depression Rating Scale; MoCA, Montreal Cognitive Assessment; LED, Levodopa equivalent dose*.

### Comparison of NMSs Between the Patients With and Without Sleep Disorders

In total, there were no differences in sex, age, disease duration, LED, motor symptoms or mH&Y stage between the patients with and without sleep disorders (Table S1). Among the patients with cognitive dysfunction, there were also no differences between those with and without sleep disorders. However, among the patients without cognitive dysfunction, those with sleep disorders had a higher UPDRS part III score, a higher mH&Y stage and a higher LED than those without sleep disorders (*P* = 0.029, *P* = 0.028, and *P* = 0.003, respectively).

NMSs were also compared between the patients with and without sleep disorders among the total patient group and in subgroups of patients with and without cognitive dysfunction. The differences in NMSs are shown in Table 2.

**Table 2:** Non-motor symptoms of enrolled patients with early-stage Parkinson's disease according to the presence/absence of sleep disorders.

	**Without sleep disorders (*N* = 297)**	**With sleep disorders (*N* = 92)**	***t***	***P*^**a**^**	**Patients with cognitive dysfunction (*****N*** **=** **155)**		***T***	***p*^**a**^**	**Patients without cognitive dysfunction (*****N*** **=** **234)**		***t***	***P*^**a**^**
					**Without sleep disorders**	**With sleep disorders**			**Without sleep disorders**	**With sleep disorders**		
					***N*** **=** **101(65.2%)**	***N*** **=** **54(34.8%)**			***N*** **=** **196(83.8%)**	***N*** **=** **38(16.2%)**		
NMS-Quest, mean ± SD	8.40 ± 4.23	13.40 ±5.08	−7.548	0.000^*^	9.07± 4.18	12.83 ± 4.87	−3.850	0.000^*^	7.84± 4.22	13.84 ± 5.28	−6.724	0.000^*^
Gastrointestinal tract symptoms, mean ± SD	1.65 ± 1.28	2.53 ± 1.51	−4.397	0.000^*^	1.80 ± 1.19	2.21 ± 1.29	−1.504	0.135	1.53 ± 1.33	2.77 ± 1.65	−4.415	0.000^*^
Urinary tract symptoms, mean ± SD	0.77 ± 0.83	1.07 ± 0.90	−2.389	0.018^*^	0.74 ± 0.82	0.96 ± 0.86	−1.180	0.240	0.80 ± 0.84	1.16 ± 0.93	−2.111	0.036^*^
Apathy/attention/memory, mean ± SD	1.46 ± 1.04	2.04 ± 1.02	−3.699	0.000^*^	1.62 ± 0.96	2.25 ± 0.90	−2.920	0.004^*^	1.33 ± 1.08	1.87 ± 1.08	−2.488	0.014^*^
Depression/anxiety/anhedonia, mean ± SD	0.70 ± 0.81	1.27 ± 0.85	−4.640	0.000^*^	0.76 ± 0.81	1.25 ± 0.90	−2.629	0.010^*^	0.65 ± 0.82	1.29 ± 0.82	−3.877	0.000^*^
Sexual function, mean ± SD	0.49 ± 0.82	0.60 ± 0.89	−0.883	0.378	0.54 ± 0.87	0.50 ± 0.89	0.220	0.829	0.44 ± 0.78	0.68 ± 0.91	−1.317	0.195
Cardiovascular symptoms, mean ± SD	0.36 ± 0.57	0.75 ± 0.70	−7.777	0.000^*^	0.36 ± 0.58	0.54 ± 0.59	−1.394	0.166	0.36 ± 0.58	0.90 ± 0.75	−4.394	0.000^*^
Hallucinations/delusions, mean ± SD	0.14 ± 0.34	0.36 ± 0.60	−2.748	0.008^*^	0.18 ± 0.39	0.54 ± 0.66	−2.557	0.017^*^	0.10 ± 0.30	0.23 ± 0.50	−1.384	0.175
Sleep, mean ± SD	1.57 ± 1.26	2.73 ± 1.25	−6.111	0.000^*^	1.83 ± 1.27	2.42 ± 1.14	−2.091	0.039^*^	1.36 ± 1.21	2.97 ± 1.30	−6.216	0.000^*^
Miscellaneous, mean ± SD	0.88 ± 0.85	1.51 ± 1.05	−4.117	0.000^*^	0.85 ± 0.79	1.71 ± 1.30	−3.086	0.005^*^	0.90 ± 0.90	1.35 ± 0.80	−2.562	0.011^*^
HAMD, mean ± SD	9.17 ± 7.07	17.62 ± 9.29	−6.317	0.000^*^	9.85 ± 6.12	15.96 ± 8.84	−3.207	0.003^*^	8.60 ± 7.75	18.90 ± 9.57	−6.306	0.000^*^
Anxiety/somatization, mean ± SD	2.19 ± 2.18	4.45 ± 2.58	−6.023	0.000^*^	2.35 ± 2.07	4.04 ± 2.69	−2.885	0.007^*^	2.05 ± 2.27	4.77 ± 2.49	−5.874	0.000^*^
Weight loss, mean± SD	0.32 ± 0.59	0.67 ± 0.82	−3.038	0.003^*^	0.33 ± 0.62	0.83 ± 0.96	−2.446	0.021^*^	0.31 ± 0.57	0.55 ± 0.68	−1.837	0.073
Cognition disorders, mean ± SD	1.01 ± 1.64	2.42 ± 2.57	−3.876	0.000^*^	0.87 ± 1.08	2.04 ± 1.90	−2.905	0.007^*^	1.12 ± 1.99	2.71 ± 2.99	−2.806	0.008^*^
Diurnal variation, mean ± SD	0.17 ± 0.40	0.38 ± 0.49	−2.947	0.004^*^	0.18 ± 0.41	0.33 ± 0.48	−1.399	0.172	0.16 ± 0.39	0.42 ± 0.50	−2.669	0.011^*^
Retardation, mean ± SD	2.16 ± 1.89	3.55 ± 2.34	−4.070	0.000^*^	2.49 ± 1.90	3.25 ± 2.40	−1.686	0.094	1.90 ± 1.86	3.77 ± 2.30	−4.210	0.000^*^
Sleep disturbances, mean ± SD	1.24 ± 1.64	3.51 ± 1.97	−7.892	0.000^*^	1.24 ± 1.58	3.14 ± 2.01	−4.820	0.000^*^	1.10 ± 1.61	3.68 ± 2.02	−7.483	0.000^*^
Hopelessness, mean ± SD	2.08 ± 1.79	2.64 ± 2.09	−2.000	0.046^*^	2.39 ± 1.82	2.38 ± 1.84	0.032	0.974	1.82 ± 1.74	2.84 ± 2.27	−2.728	0.007^*^
HAMA, mean ± SD	7.64 ± 5.84	15.49 ± 7.58	−7.181	0.000^*^	8.39 ± 5.51	14.96 ± 8.51	−3.610	0.001^*^	7.02 ± 6.04	15.90 ± 6.90	−7.117	0.000^*^
PDSS, mean ± SD	128.67 ±12.26	90.98 ±11.11	20.812	0.000^*^	125.85 ±13.12	92.92 ±10.69	11.437	0.000^*^	131.02 ±11.02	89.48 ±11.37	18.648	0.000^*^
MoCA, mean ± SD	25.79± 2.65	23.85 ± 2.35	4.969	0.000^*^	23.47± 1.85	23.04 ± 1.55	1.165	0.251	27.73± 1.33	24.48 ± 2.68	6.532	0.000^*^
Visuospatial skills/executive function, mean ± SD	3.77 ± 1.21	3.49 ± 1.17	1.522	0.129	3.05 ± 1.22	2.88 ± 1.26	0.610	0.546	4.36 ± 0.82	3.97 ± 0.84	2.391	0.018^*^
Naming, mean ± SD	2.79 ± 0.53	2.56 ± 0.60	2.595	0.011^*^	2.61 ± 0.68	2.67 ± 0.57	−0.370	0.712	2.94 ± 0.29	2.48 ± 0.63	3.983	0.000^*^
Attention, mean ± SD	5.59 ± 0.69	5.09 ± 0.89	3.864	0.000^*^	5.38 ± 0.77	5.29 ± 0.75	0.501	0.617	5.76 ± 0.56	4.94 ± 0.96	4.562	0.000^*^
Language, mean ± SD	2.63 ± 0.64	2.60 ± 0.60	0.268	0.789	2.37 ± 0.77	2.50 ± 0.66	−0.773	0.441	2.84 ± 0.41	2.68 ± 0.54	1.552	0.129
Abstraction, mean ± SD	1.68 ± 0.56	1.62 ± 0.59	0.705	0.481	1.46 ± 0.68	1.29 ± 0.69	1.062	0.290	1.86 ± 0.35	1.87 ± 0.34	−0.117	0.907
Delayed memory, mean ± SD	3.39 ± 1.43	2.98 ± 1.23	2.131	0.036^*^	2.68 ± 1.52	2.54 ± 1.32	0.409	0.683	3.98 ± 1.03	3.32 ± 1.05	3.145	0.002^*^
Orientation, mean ± SD	5.95 ± 0.25	5.49 ± 0.69	4.869	0.000^*^	5.91 ± 0.35	5.88 ± 0.34	0.482	0.631	5.98 ± 0.13	5.19 ± 0.75	5.853	0.000^*^

SD, standard deviation; PD, Parkinson's disease; NMS-Quest, Non-Motor Symptoms Questionnaire; HAMD, Hamilton Depression Rating Scale; HAMA, Hamilton Anxiety Rating Scale; PDSS, The Parkinson's Disease Sleep Scale; MoCA, Montreal Cognitive Assessment.

a The P-value is calculated adjusted for age, disease duration, UPDRS part III score, mH&Y stage, and LED.

* *Significant difference*.

In total, the patients with sleep disorders had significantly higher scores on the subdomains of the NMS-Quest scale than those without sleep disorders, including gastrointestinal symptoms, urinary tract symptoms, apathy/attention/memory, depression/anxiety/anhedonia, cardiovascular symptoms, hallucinations/delusions, and miscellaneous. There were also significant differences in the total HAMD and HAMA scores between the groups, and the scores for all 7 domains of the HAMD were higher in the patients with sleep disorders than in those without sleep disorders (all *P* < 0.05). The patients with sleep disorders obtained worse scores on the MoCA overall and in the domains of naming, attention, delayed memory and orientation compared with the patients without sleep disorders (all *P* < 0.05).

Among the patients with cognitive dysfunction, those with sleep disorders had significantly higher scores on the NMS-Quest subdomains, including apathy/attention/memory, depression/anxiety/anhedonia, hallucinations/delusions, and miscellaneous. The analysis of the HAMA scores indicated that anxiety symptoms were more common in patients with sleep disorders (*P* = 0.001). There were also significant differences in the total HAMD scores, and the scores on the anxiety/somatization, weight loss, and cognition disorders domains of the HAMD were higher in the patients with sleep disorders than in those without sleep disorders. There were no significant differences in total MoCA or the MoCA domain scores between the groups (all *P* < 0.05).

Among the patients without cognitive dysfunction, after controlling for the effects of the UPDRS part III score, mH&Y stage, and LED, the patients with sleep disorders had significantly higher scores on the NMS-Quest subdomains, including gastrointestinal symptoms, urinary tract symptoms, apathy/attention/memory, depression/anxiety/anhedonia, cardiovascular symptoms, and miscellaneous (all *P* < 0.05). The scores of the HAMD and subdomains of anxiety/somatization, cognition disorders, diurnal variation, retardation, and hopelessness were higher in patients with sleep disorders (all *P* < 0.05). The HAMA score indicated that anxiety symptoms were more common in the patients with sleep disorders (*P* < 0.05). Those patients also obtained relatively worse scores on the MoCA overall and in the domains of visuospatial/executive skills, naming, attention, delayed memory and orientation (all *P* < 0.05).

### Comparison of Sleep Parameter Between Patients With and Without Cognitive Dysfunction

A higher percentage of patients with cognitive dysfunction than patients without cognitive dysfunction had sleep disorders (34.8 vs. 16.2%, *P* < 0.01). Compared with patients without cognitive dysfunction, patients with cognitive dysfunction also had lower PDSS scores and SE, a longer NREM sleep stage 1 sleep time (N1), a shorter NREM sleep stage 3 sleep time (N3), a shorter total sleep time, a longer SL and a longer WASO (all *P* < 0.05). The AHI and PLMI scores were not different between the two groups (both *P* > 0.05) (Table S2).

### NMSs Independently Correlated With Sleep Disorders

The results of the multivariate logistic regression analysis are summarized in Table 3. In the total sample, sleep disorders were related to higher scores on the NMS urinary tract subdomain (OR 1.657, 95% CI, 1.060–2.590) and the hallucinations/delusions subdomain (OR 1.966, 95% CI, 1.072–3.606). The HAMD hopelessness subscale score (OR 1.577, 95% CI, 1.151–2.161) and HAMA score (OR 1.240, 95% CI, 1.154–1.332) were also significant determinants of sleep disorders. Better cognitive performance on the MoCA orientation subdomain (OR 0.124, 95% CI, 0.053–0.288) may predict better sleep quality.

**Table 3:** Clinical factors correlated with sleep quality in the total sample of PD patients.

	***B***	***S.E*.**	***Wals***	***Df***	***OR***	**95% *CI***	***P*^**a**^**
NMS-Urinary tract symptoms	0.562	0.413	5.932	1	1.657	1.060–2.590	0.027
NMS-Hallucinations/delusions	0.814	0.231	3.876	1	1.966	1.072–3.606	0.029
HAMD-Hopelessness	0.651	0.325	4.357	1	1.577	1.151–2.161	0.025
HAMA	0.225	0.038	35.777	1	1.240	1.154–1.332	0.000
MoCA-Orientation	−2.155	0.440	23.996	1	0.124	0.053–0.288	0.015

S.E., standard error; Df, degree of freedom; OR, odds ratio; CI, confidence interval; NMS, Non-Motor Symptoms Questionnaire; HAMD, Hamilton Depression Rating Scale; HAMA, Hamilton Anxiety Rating Scale; MoCA, Montreal Cognitive Assessment.

a *The P-value was calculated using a forward binary logistic regression analysis*.

In the patients with cognitive dysfunction, sleep disorders were related to higher scores on the NMS hallucinations/delusions subdomain (OR 3.860 95% CI, 1.397–10.663) and the HAMD weight loss subdomain (OR 1.995, 95% CI, 1.020–3.900). The HAMA score (OR 1.148, 95% CI, 1.058–1.245) was also a significant determinant of sleep disorders (Table 4).

**Table 4:** Clinical factors correlated with sleep quality in PD patients with cognitive dysfunction.

	***B***	***S.E***	***Wals***	***Df***	***OR***	**95% *CI***	***P*^**a**^**
NMS-Hallucinations/delusions	1.167	0.533	4.804	1	3.860	1.397–10.663	0.009
HAMA	0.522	0.116	20.402	1	1.148	1.058–1.245	0.001
HAMD-weight loss	0.875	0.312	7.842	1	1.995	1.020–3.900	0.000

S.E., standard error; Df, degree of freedom; OR, odds ratio; CI, confidence interval; NMS, Non-Motor Symptoms Questionnaire; HAMD, Hamilton Depression Rating Scale; HAMA, Hamilton Anxiety Rating Scale.

a *The P-value was calculated using a forward binary logistic regression analysis*.

In the patients without cognitive dysfunction, the multivariate logistic regression analysis indicated that sleep disorders were related to higher HAMA scores (OR 2.207, 95% CI, 1.210–4.027) and higher LED (OR 1.957, 95% CI, 1.384–2.768). Better cognitive performance on the MoCA naming (OR 0.427, 95% CI, 0.265–0.687) and MoCA orientation (OR 0.155, 95% CI, 0.048–0.503) subdomains may predict better sleep quality (Table 5).

**Table 5:** Clinical factors correlated with sleep quality in PD patients without cognitive dysfunction.

	***B***	***S.E***	***Wals***	***Df***	***OR***	**95% *CI***	***P*^**a**^**
LED	0.671	0.177	14.403	1	1.957	1.384–2.768	0.000
HAMA	0.792	0.307	6.659	1	2.207	1.210–4.027	0.000
MoCA-Naming	−0.851	0.243	12.264	1	0.427	0.265–0.687	0.000
MoCA-Orientation	−1.861	0.599	9.668	1	0.155	0.048–0.503	0.000

S.E., standard error; Df, degree of freedom; OR, odds ratio; CI, confidence interval; LED, levodopa equivalent dose; HAMA, Hamilton Anxiety Rating Scale; PDSS, Parkinson's Disease Sleep Scale; MoCA, Montreal Cognitive Assessment.

a *The P-value was calculated using a forward binary logistic regression analysis*.

## Discussion

In PD, motor symptoms and several other factors are intrusive in daily life and increase sleep disturbances. This study aimed to assess the impact of NMSs and cognitive status on sleep quality.

Sleep disturbance is one of the most common NMSs in PD. It has received significant attention in recent years, but it is often underrecognized and undertreated in clinical practice. The 15-item PD Sleep Scale (PDSS) was developed as a reliable and specific clinical tool for sleep self-evaluation in PD and as a screening tool and a measure of severity recommended by the Sleep Scale Task Force of the Movement Disorder Society (17, 23, 24). In our study, in addition to subjective scale assessments, we also included objective PSG to obtain more accurate results. In our sample, approximately 23.7% of the patients suffered from sleep disorders, which suggests that approximately one-quarter of PD patients suffer from sleep disturbances. This finding is in accordance with results obtained in several previous surveys (18, 25). The mean total PDSS score in our study was 121.3, and a similar score was reported by Wang et al. (118.4) (26), although lower total scores have been reported in most Western populations (17, 27, 28). The variability in the prevalence of sleep disturbances and total PDSS scores is most likely due to differences in study populations and methodologies.

In our study, statistically significant correlations were observed between sleep quality and NMS symptoms across all patients, consistent with previously published studies (28–31). The PDSS scores were associated with the MoCA, HAMD and NMS scores along several dimensions, thus confirming the first hypothesis regarding a relationship between sleep quality and the presence and severity of NMSs. We observed that the PD patients with poor sleep quality who experienced all domains of depressive symptoms had significantly worse sleep quality and higher rates of sleep disturbance than the patients without these symptoms. Clinical features were entered in a multiple logistic regression model to identify the factors that independently affected sleep quality. We found that the NMS urinary tract symptoms subdomain score was an independent risk factor for the development of poor sleep quality in patients with early-stage PD. Urinary symptoms may be related to autonomic dysfunction, although some evidence suggests that nigrostriatal dopamine may exert direct control over bladder function. Urinary symptoms are common NMSs that cause discomfort and an unpredictable need to use the bathroom at night and may contribute to disrupted sleep. Our findings confirm that depression and anxiety are the main determinants of sleep quality, which is in line with several previous studies that reported that depression was the main determinant of sleep disorders (32–35). The effects of these symptoms on sleep in PD patients have not been fully examined. Disrupted regulation of neurotransmitters such as norepinephrine, serotonin, and dopamine and pathological changes in brainstem nuclei may be potential common mechanisms of both mood control and circadian rhythms (36). To the best of our knowledge, our study is the first to discover that cognitive function, especially orientation, affects sleep quality in the early stage of PD. Possible mechanisms underlying this comorbidity require further study.

In patients with cognitive dysfunction, we found that the NMS hallucinations/delusions score was the most important NMS determinant of sleep disorders. Minor hallucinations were reported in 42% of patients with very early disease (37). Symptoms on the psychosis spectrum in the early stages of PD include minor experiences, such as passage and presence hallucinations, illusions, and formed hallucinations (38). Several studies have suggested that cognitive dysfunction, hallucinations and sleep are related phenomena in PD (39, 40). Our previous study (41) showed that patients with visual hallucinations had relatively worse sleep quality, which is consistent with previous studies showing that PD patients with hallucinations had reduced sleep efficiency and increased awakenings compared to PD patients without hallucinations (42). That study also showed that better cognition, especially in domains such as attention and orientation, may protect against hallucinations (41). The results of this study confirmed the relationship among hallucinations, cognition and sleep quality in early-stage PD.

In the patients without cognitive dysfunction, we found that a higher LED was an independent determinant of the risk of sleep disorders. Both the dose and timing of dopaminergic medications may influence sleep quality in PD patients. However, this relationship has not been systematically studied in early-stage PD. The effects of dopaminergic medications on specific sleep stages are also poorly understood. Although levodopa coverage at night can improve sleep in PD patients, ongoing or elevated levodopa therapy may also be associated with neuropsychiatric symptoms, which could precipitate sleep disturbances. Patients with early PD experienced fewer sleep disturbances when treated with rasagiline compared to pramipexole (43). Elucidating the effects of dopaminergic medications on sleep will be important for identifying strategies to optimize sleep in PD patients. It is important to note that optimized treatment to improve sleep quality paralleled improvement in nocturnal motor symptoms.

This study has some limitations: (1) There was no age- and sex-matched healthy population for comparison; (2) The cross-sectional design prevented causal inferences. Information from patients in more advanced stages or with dementia may have biased the data; (3) The sleep data were based on sleep quality self-evaluations and on a gold-standard measurement of sleep architecture, PSG. However, in this study, we did not take full advantage of the objective parameters of PSG; (4) Additional studies are needed to more thoroughly explore what type of sleep disorders might be influenced by NMSs.

## Conclusions

In conclusion, we found that poor sleep quality had a prevalence of 23.7% in early-stage PD patients. Our results confirmed the high prevalence of cognitive dysfunction in patients with PD (39.8%). The patients with cognitive dysfunction suffered a higher percentage of sleep disorders, lower SE and longer SL and WASO. In total, the patients who suffered from NMSs, such as depressive symptoms, anxiety symptoms, urinary tract symptoms, and hallucinations/delusions, had relatively worse sleep quality. Better cognition may predict better sleep quality. Among the patients with cognitive dysfunction, the NMS hallucinations/delusions score was the most important risk factor for sleep disorders. Among the patients without cognitive dysfunction, NMSs such as anxiety and cognition and medication were related to sleep disorders. Future studies should focus on elucidating the pathophysiology of these symptoms.

## Data Availability Statement

The datasets generated for this study are available on request to the corresponding author.

## Ethics Statement

The studies involving human participants were reviewed and approved by the Ethics Committee of the Nanjing Brain Hospital Affiliated with Nanjing Medical University. The patients/participants provided their written informed consent to participate in this study.

## Author Contributions

JZ and LiZ participated in conception and design of the study, acquisition of raw data, analysis of data, drafting the article, and revising the manuscript. MZ, ZW, YP, and BS participated in conception and design of the study, collection of date, analysis of data, and revising the manuscript. JY, XJ, LilZ, and JD participated in conception and design of the study, and revising the manuscript.

## Conflict of Interest

The authors declare that the research was conducted in the absence of any commercial or financial relationships that could be construed as a potential conflict of interest.
