# Supplementary material for: Nonmotor Symptoms Affect Sleep Quality in Early-Stage Parkinson's Disease Patients With or Without Cognitive Dysfunction
Source: Front Neurol. 2020 Apr 21;11:292. doi: 10.3389/fneur.2020.00292 (PMC7186472; doi:10.3389/fneur.2020.00292)
Supplement: Supplementary file 1 [file Data_Sheet_1.pdf]

Table S1. Demographic characteristics of enrolled patients with early-stage Parkinson’s disease according to the presence/absence of sleep disorders.

	Without sleep disorders (N=297)	With sleep disorders （N=92）	t/x^2	*P*	Patients with cognitive dysfunction (N=155)		t/x^2	*p*	Patients without cognitive dysfunction (N=234)		t/x^2	*P*
					Without sleep disorders	With sleep disorders			Without sleep disorders	With sleep disorders		
					N=101(65.2%)	N= 54(34.8%)			N=196(83.8%)	N= 38(16.2%)		
Sex (male, %)	193(65.0%)	54(58.7%)	1.198	0.173 [b]	68(67.3%)	33(61.1%)	0.599	0.357 [b]	125(63.8%)	21(55.3%)	0.982	0.283 [b]
Age, y, mean ± SD	63.92 ± 8.84	63.75 ± 9.09	-0.120	0.905 [a]	64.79±8.79	63.42±11.05	-0.670	0.504 [a]	63.20±8.86	64.03±9.08	0.465	0.643 [a]
Duration, y, mean ± SD	3.12 ± 3.64	3.51 ± 3.26	1.943	0.056 [a]	3.05 ± 3.45	3.61 ± 3.23	1.546	0.133 [a]	2.99 ± 3.80	3.73 ± 3.60	1.304	0.194 [a]
UPDRS-III, mean ± SD	17.73 ± 9.07	19.86 ± 9.36	1.570	0.117 [a]	18.42 ± 8.92	18.27 ± 10.15	-0.073	0.942 [a]	17.18 ± 9.19	21.19 ± 8.59	2.204	**0.029** [a]*
mH&Y stage, mean ± SD	1.68 ± 0.54	1.78 ± 0.55	1.192	0.234 [a]	1.71 ± 0.55	1.64 ± 0.56	-0.601	0.549 [a]	1.67± 0.54	1.90 ± 0.52	2.220	**0.028** [a]*
LED, mg, mean ± SD	334.71 ± 309.17	386.89 ± 382.03	1.053	0.561 [a]	346.28 ± 314.21	351.71 ± 323.42	0.207	0.837 [a]	310.44 ± 291.45	503.15 ± 389.83	3.064	**0.003** [a]*

SD: standard deviation. PD: Parkinson’s disease. UPDRS-III: Unified Parkinson’s Disease Rating Scale part III. mH&Y stage: modified Hoehn and Yahr stage

[a] Student's t-test

[b] Chi-square test

* Significant difference

Table S2. Sleep parameters of enrolled patients with early-stage Parkinson's disease according to the presence/absence cognitive dysfunction.

	Patients with cognitive dysfunction (N=155)	Patients without cognitive dysfunction (N=234)	t/x^2	*P*
PDSS, mean ± SD	101.23 ± 13.29	129.35 ± 14.19	4.615	**0.000** [a*]
Sleep lantency, min, mean ± SD	52.34 ± 18.26	19.47 ± 9.12	-3.597	**0.000** [a*]
Wake after sleep onset, min, mean ± SD	76.23 ± 28.17	39.27 ± 22.49	-5.432	**0.000** [a*]
Sleep efficiency, %	78.65 ± 32.87	90.36 ± 42.62	6.376	**0.000** [a*]
N1 time (min), mean ± SD	41.93 ± 35.86	22.44 ±18.73	-4.543	**0.000** [a*]
N3 time (min), mean ± SD	43.25 ± 28.32	71.06 ±48.47	7. 735	**0.000** [a*]
Total sleep time (min), mean ± SD	342.41 ± 106.28	429.14 ± 111.47	6.563	**0.000** [a*]
AHI, mean ± SD	6.11 ± 5.62	5.21 ± 6.59	-0.341	0.403 [a]
PLMI, mean ± SD	10.23 ± 9.62	9.78 ± 7.28	-0.564	0.874 [a]
With sleep disorders	54(34.8%)	38(16.2%)	17.863	**0.000** [b*]
Without sleep disorders	101(65.2%)	196(83.8%)		

SD: standard deviation. PDSS: Parkinson's Disease Sleep Scale. N1 time: NREM sleep stage 1 sleep time. N3 time: NREM sleep stage 3 sleep time.

AHI: apnea-hypopnea index. PLMI: periodic limb movement index

[a] Student's t-test

[b] Chi-square test

* Significant difference
